# Oxidative Stress Response of Liver Cell Culture in Atlantic Salmon Challenged Under Two Antibiotics: Oxytetracycline and Florfenicol

**DOI:** 10.3390/toxics13050361

**Published:** 2025-04-30

**Authors:** Luis Vargas-Chacoff, Francisco Dann, Ricardo Oyarzún-Salazar, Daniela Nualart, José Luis P. Muñoz

**Affiliations:** 1 Laboratorio de Fisiología de Peces, Instituto de Ciencias Marinas y Limnológicas, Universidad Austral de Chile, Valdivia 5090000, Chile; franciscojavierdann@gmail.com (F.D.); daniela.nualart@gmail.com (D.N.); 2 Millennium Institute Biodiversity of Antarctic and Subantarctic Ecosystems (BASE), University Austral of Chile, Valdivia 5090000, Chile; 3 Centro FONDAP de Investigación en Dinámica de Ecosistemas Marinos de Altas Latitudes (IDEAL), Universidad Austral de Chile, Valdivia 5090000, Chile; 4 Integrative Biology Group, Universidad Austral de Chile, Valdivia 5090000, Chile; 5 Laboratorio Institucional de Investigación, Facultad de Ciencias de la Naturaleza, Universidad San Sebastián, Puerto Montt 5480000, Chile; ricardo.oyarzun@uss.cl; 6 Programa de Doctorado en Ciencias de la Acuicultura, Escuela de Graduados, Universidad Austral de Chile, Puerto Montt 5480000, Chile; 7 Centro de Investigación y Desarrollo I~Mar, Universidad de los Lagos, Puerto Montt 5480000, Chile

**Keywords:** antibiotics, cell culture, stress, aquaculture, salmon

## Abstract

Aquaculture is currently the fastest-growing sector in animal production, with an average annual growth rate of 7.5% since 1970. In Chile, the industry is largely driven by salmonid farming, with *Salmo salar* (Atlantic salmon) accounting for over 65% of national production. This species has shown the most significant growth within the sector. This growth is achieved by having high-density farming, which results in high levels of stress due to overcrowding and the appearance of pathogens such as the Infectious Salmon Anemia (ISA) virus, Bacterial Kidney Disease (BKD), Caligus sea lice (*Caligus rogercresseyi*), and Piscirickettsiosis (SRS) caused by *Piscirickettsia salmonis*, among others. This study evaluated the toxicity of the two most commonly used antibiotics in the salmon industry—oxytetracycline and florfenicol—at four concentrations each, using primary liver cell cultures of Atlantic salmon (Salmo salar). Oxidative stress was assessed through enzymatic activity and gene expression of oxidative markers, including cytochrome P450, catalase (CAT), superoxide dismutase (SOD), glutathione reductase (GR), and glutathione peroxidase (GPx). Samples were analyzed at 1, 3, 6, 12, and 48 h post-exposure. These findings reveal time- and dose-dependent oxidative responses in salmon liver cells to OTC and FLO, providing critical insights into the sublethal cellular effects of antibiotics commonly used in aquaculture, which indicates the presence of a high amount of free radicals in the liver cells, indicating toxicity of both antibiotics.

## 1. Introduction

Aquaculture is currently the fastest-growing animal production industry, with an annual increase of 7.5% from 1970 to the present [1]. In Chile, aquaculture is dominated by salmonid production. *Salmo salar* (Atlantic salmon) accounts for 65% of Chilean production, with the culture of this species experiencing the highest growth. This growth is achieved by having the culture in high densities, which results in high levels of stress due to overcrowding and the appearance of pathogens such as Infectious Salmon Anaemia (ISA) virus, Bacterial Kidney Disease (BKD), caligus sea louse (*Caligus rogercresseyi*), and Piscirickettsiosis (SRS) caused by *Piscirickettsia salmonis*, among others [2,3,4].

To reduce the high mortality rates caused by these pathogens, the industry has promoted the use of antiparasitic medications and antibiotics. Florfenicol and oxytetracycline are the most commonly used [5,6]. In the case of florfenicol, its chemical structure shows that it is a synthetic fluorinated analog derived from Chloramphenicol with bacteriostatic action, which has properties against gram-negative and gram-positive bacteria. It is widely used to treat livestock and aquaculture pathogens [7,8]. Oxytetracycline is a broad-spectrum antibiotic used in fish culture, is low-cost, has bacteriostatic action against gram-negative and gram-positive bacteria, and belongs to the tetracycline family [9,10,11].

Numerous studies confirm that the excessive use of antibiotics in animals increases the formation of reactive oxygen species (ROS), causing the antioxidant system to be unable to tolerate the large amount of free radicals in the body. The effect of this excessive production of ROS, and/or the inability of the antioxidant defense systems to counteract them, is known as oxidative stress [7,9,12,13]. Oxidative stress occurs when there is an imbalance between the amount of reactive oxygen species (ROS) in the body and the ability of antioxidants to neutralize them. ROS are a group of molecules that contain oxygen and are highly unstable because they have an unpaired electron in their conformation. These molecules are known as free radicals, and they have a high capacity to react with other molecules. These are produced by physiological aerobic metabolism under stable conditions in which the organism can tolerate this imbalance using antioxidants such as Catalase (CAT), Glutathione peroxidase (GPx), Glutathione reductase (GR), and Superoxide dismutase (SOD) [14].

Catalase reduces oxidative stress by transforming hydrogen peroxide (H_2_O_2_) into water and oxygen. Meanwhile, GPx and GR operate together, with GR facilitating the conversion of oxidized glutathione (GSSG) back to its reduced form (GSH), which GPx then utilizes to reduce hydrogen peroxide and lipid hydroperoxides in the plasma membrane. SOD is responsible for dismutating the superoxide radical (O_2_^−^) into hydrogen peroxide (H_2_O_2_) and oxygen, thus taking care of one of the major ROS radicals [15,16].

Another system involved in the body’s defence against excess antibiotics is the xenobiotic detoxification system, formed by the cytochrome P450 superfamily of proteins. Its function is to detoxify any foreign chemical not synthesized by the organism, transforming it into a soluble compound, thus facilitating its elimination [17,18]. This detoxification system is divided into two phases. Phase I involves the oxidation, reduction, or hydrolysis of the xenobiotic by polar functional groups, such as carboxyls, amines, and hydroxyls. In Phase II, a series of transferases intervene in the reactions generated in Phase I (xenobiotics or metabolites), employing conjugation reactions that increase water solubility to facilitate their excretion through urine or bile [19,20].

The objective of this study is to determine the oxidative response through the determination of enzymatic antioxidants previously mentioned, such as “SOD, GPx, GR, and Catalase”, as well as protein complexes involved in the biotransformation of xenobiotics such as cytochrome P450, both at enzymatic and gene expression levels in the cell culture of Atlantic Salmon liver challenged with the most used antibiotics, such as Florfenicol and Oxytetracycline.

## 2. Materials and Methods

### 2.1. Ethics Statement

All experimental procedures complied with regulations for laboratory animal use established by the Chilean National Commission for Scientific and Technological Research (ANID) and the University of Los Lagos (Res. # 01/2023).

### 2.2. Sampling Procedure

Healthy juvenile specimens of *Salmo salar* (n = 6) (mean weight = 60 ± 10 g) were obtained from the Fish Nutrition and Physiology Laboratory of the Catholic University of Temuco and then transferred to the facilities of the Calfuco Coastal Aquatic Resources Laboratory belonging to the Austral University of Chile, where they were acclimatized for 2 weeks in 200 L tanks at room temperature (12 ± 1 °C), in fresh water, using a continuous flow system with natural photoperiod. They were fed daily with dry commercial pellets for salmonids (BioMar,, Puerto Montt, Chile, Golden optima 5222, containing 50.5% protein, 20.5% lipids, 1.2% crude fiber, 10% moisture, and 12% ash). For the extraction of liver tissue, the fish were caught with a net and sedated using a lethal dose of 2-phenoxyethanol (1 mL/L), following the protocol of Vargas-Chacoff et al. [4].

### 2.3. Primary Culture

For primary culture, small pieces of liver tissue (approximately 15–20 mg) were obtained from *Salmo salar* liver explants and removed under aseptic conditions [21]. The liver was used for primary cell culture, which was seeded, maintained in a six-well plate, and cultured at 18 °C under an air atmosphere for at least 24 h [21]. The medium used for cell and tissue culture was Leibovitz’s 15 (L-15), with each well containing 1 mL of L-15. This medium was improved by adding 10% fetal bovine serum (FBS) from Invitrogen (Gibco, Thermo Fisher, Waltham, MA, USA), following the protocols of Pontigo and Vargas-Chacoff [22] and Nualart et al. [23]. For kinetic experiments at 1, 3, 6, 12, 24, and 48 h at 18 °C, 1 μL/well of the antibiotic solution was added. Control plates had the same volume of medium without the antibiotic. All experiments were conducted in triplicate and repeated independently two times.

### 2.4. In Vitro Experimental Treatment

Twenty-four hours after seeding each portion of the liver, the medium was changed for testing at different doses of oxytetracycline (OTC) (Merck, Darmstadt, Germany; CAS No: 79–57-2): 0.25 µg/mL, 0.5 µg/mL, 1.5 µg/mL, 3 µg/mL, and a control group. Different doses of florfenicol (FLO) (Merck; CAS No: 79–57-2)—1 µg/mL, 4 µg/mL, 10 µg/mL, and 20 µg/mL—were also evaluated. The doses of OTC selected are akin to those outlined by Tafalla et al. [24]. The FLO doses were chosen based on the work of Martinsen et al. [25]. Subsequently, the doses applied were derived from a pharmacokinetic study of OTC and FLO in Atlantic salmon residing in seawater, using antibiotic plasma levels as a reference [25,26].

### 2.5. Extraction of Total RNA from Liver Cells

After completing the kinetic experiments, the cell culture medium should be eliminated, and 500 μL of TRIzol reagent (Sigma, Kawasaki, Japan) should be introduced to collect the cells. Subsequently, these cells are frozen in liquid nitrogen for RNA extraction. Using TRIzol reagent, total RNA was extracted following the manufacturer’s instructions and stored at −80 °C. The RNA concentration was assessed at a wavelength of 260 nm using a NanoDrop spectrophotometer from NanoDrop Technologies^®^ (Waltham, MA, USA). A 1% agarose gel electrophoresis was performed to assess the RNA sample’s quality. Ultimately, 2 μg of RNA was utilized as a template for the reverse transcription process to create cDNA. Following standard protocols, this procedure employed the MMLV-RT reverse transcriptase supplied by Promega (Madison, WI, USA) and the oligo-dT primer from Invitrogen.

### 2.6. qPCR Analysis

The experiments were carried out using the AriaMx Real-time PCR System from Agilent, Santa Clara, CA, USA. The cDNA was prepared at a concentration of 100 ng and used as the template for qRT-PCR with Brilliant SYBRGreen qPCR reagents from Stratagene, La Jolla, CA, USA. Each reaction was conducted in triplicate with a total volume of 14 μL, comprising 6 μL of SYBRGreen, 2 μL of cDNA (at 100 ng), 1.08 μL of a primer mixture, and 4.92 μL of PCR-grade water. The PCR protocol included an initial step at 95 °C for 10 min, followed by 40 cycles of 95 °C for 10 s, 60 °C for 15 s, and 72 °C for 15 s. Post-PCR, a melting curve analysis was performed on the amplified products to ensure that only a single PCR product was amplified and detected. Gene expression levels of Cytochrome-P450 (P450), glutathione reductase (GR), glutathione peroxidase (GPx), and superoxide dismutase (SOD) were assessed using the comparative Ct method (2^−ΔΔCT^) [27]. The findings are expressed as fold changes in gene expression, standardized using the endogenous reference gene 18S, and contrasted with control cells that were not stimulated. The primers used for this work are found in Table 1, and their efficiency was calculated using the Rasmussen formula. Table 1 provides the specific primers [28].

### 2.7. Antioxidant Enzyme Activity

#### Homogenization

The liver was homogenized in a 100 mM phosphate buffer at pH 7.4, which contained 0.15 M KCl, 1 mM EDTA, 0.1 mM PMSF, and 1 mM DDT in a 1:4 ratio (sample weight to volume buffer). The samples were centrifugated at 12,000 rpm for 30 min, with the temperature held at 4 °C. The resulting supernatants were then utilized to assess the enzymatic activity. These measurements were conducted in triplicate using a spectrophotometric analysis with a microplate reader (MultiscanGo, Thermo Scientific) and analyzed with ScanIT 3.2 MultiscanGo software.

Enzymatic activities were measured at 20 °C and expressed in substrate moles converted to product per minute. Specific enzymatic activities are expressed according to the protein concentration in each sample (mU/mg or U/mg).

### 2.8. Specific Enzymatic Conditions

Catalase (CAT) activity was assessed following the methodologies described by Aebi [29] and López-Galindo et al. [30]. The rate at which the H_2_O_2_ substrate is broken down was observed at 240 nm for 5 min. The findings are reported with the understanding that one unit of CAT activity represents the amount of H_2_O_2_ molecules degraded per minute per milligram of protein.

Glutathione Reductase (GR) activity is responsible for facilitating the conversion of oxidized glutathione (GSSG) back to its reduced form (GSH), a process aided by the enzyme glutathione peroxidase (GPx), which plays a critical role in reducing peroxides and lipid hydroperoxides. This activity is quantified in terms of mmol/min/mg protein. It was performed at 340 nm over 5 min, monitoring the oxidation of NADPH following the procedures laid out by Carlberg and Mannervik [31] and López-Galindo et al. [30].

Glutathione Peroxidase (GPx) activity involves the catalysis of converting hydrogen peroxide and lipid hydroperoxides into less harmful substances by utilizing reduced glutathione (GSH) as a reducing agent. This process is assessed by measuring the oxidation of NADPH at an absorbance of 340 nm, as outlined in the studies by Flohe and Gunzler [32] and López-Galindo et al. [30].

Superoxide Dismutase (SOD) activity focuses on the interaction between xanthine and xanthine oxidase, which generates the superoxide anion (O_2_^−^). SOD then facilitates the conversion of this anion into hydrogen peroxide (H_2_O_2_) and oxygen. The method, as described by Sun et al. [33], determines the activity of superoxide dismutase (SOD) based on its capacity to inhibit the transformation of nitroblue tetrazolium (NBT) into formazan blue. This inhibition is measured at a wavelength of 560 nm over 30 min.

**Table 1:** Primer sequences for expression analysis.

Primer	Nucleotide Sequences (5′ → 3′)	Efficiency (%)	GenBank No/Reference
P450Fw	TCGTTCCTTGTCCGAAAGCAGA	100.4	Pedro et al., 2019 [34]
P450 Rv	TGTCGGTACCAGCACCAAACAT		
GR Fw	AAAGTGCCAGTACCAAGCCC	101.7	Martinez et al., 2018 [35]
GR Rv	CATGCTGATGAGCTACTGTTGTT		
SOD Fw	GGGCAATGCCAATAACTCCACA	104.5	Pedro et al., 2019 [34]
SOD Rv	AGGACCATGGTGATCCATGAGAAG		
GPx Fw	GAACTGCAGCAATGGTGAGA	100.3	Pedro et al., 2019 [34]
GPx Rv	CATGAGAGAGATGGGGTCGT		
18S Fw	GTCCGGGAAACCAAAGTC	103.5	Pedro et al., 2019 [34]
18S Fw	TTGAGTCAAATTAAGCCGCA		

### 2.9. Protein Quantification

This was conducted using the bicinchoninic acid method, employing the BCA Protein Assay Kit (Pierce #23225, Darmstadt, Germany). All assessments were executed using a MultiscanGo Microplate reader (Thermo Scientific) and ScanIT 3.2 software.

### 2.10. Statistical Analyses

All the data are shown as the average ± standard error of the mean (S.E.M.). The efficiency of PCR was determined by performing linear regression analysis on the sample data using LinRegPCR (Microsoft #365, WA, USA). A two-way ANOVA was used to analyze gene expression, considering different stimuli and time as factors contributing to variance. Prior to this, the data were examined to ensure normality and homoscedasticity. Following the ANOVA, Tukey’s HSD test was conducted as a post hoc analysis.

## 3. Results

### 3.1. Oxytetracycline (OTC)

**Figure 1:**
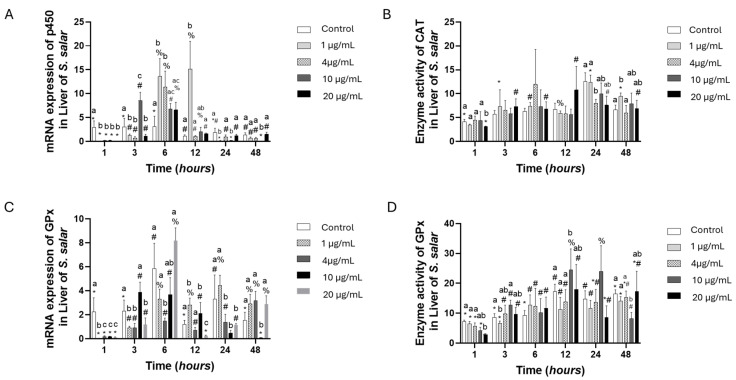
Liver cell culture treated with oxytetracycline “OTC”: gene expression with (**A**) Citochrome p450 and (**C**) GPx, and enzyme activity with (**B**) CAT and (**D**) GPx. The relative expression of genes was calculated by the comparative Ct method (2^−ΔΔCT^) using the 18s ribosomal protein as the internal reference gene. Each value represents the mean ± S.E.M. Letters indicate statistical differences among different treatments at the same time, statistical differences over time points. Symbols (*, #, and %) indicate statistical differences among the same treatments at different time points (two-way ANOVA, *p* < 0.05).

The gene expression of p450 presented downregulation at 1 h concerning the control group. However, at 6 h of exposure, the expression was overexpressed in all concentrations, with the highest levels at 6 and 12 h at 1 µg/mL of OTC; after this, the expression decreased. Catalase enzyme activity presented low expression at 24 h at 4, 10, and 20 µg/mL of OTC (Figure 1A,B). Concurrently with this, the activity of the catalase enzyme was observed to be low at 24 h when exposed to 4, 10, and 20 µg/mL of OTC (Figure 1A,B). The GPx gene expression presented downregulation compared to the control group at almost all concentrations and times, except for some concentrations that were similar to the expression of the control group. However, no concentration was significantly higher than the control group. The GPx enzyme activity at 1 h of the challenge showed decreased activity in all experimental groups; only 20 µg/mL of OTC was statistically significant. The rest of the kinetic levels were similar, but at 12 h, 10 µg/mL of OTC was higher than the control group (Figure 1C,D).

**Figure 2:**
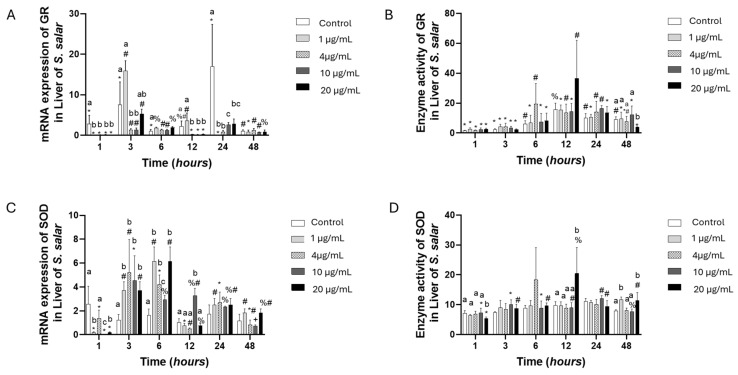
Liver cell culture treated with oxytetracycline “OTC”: gene expression with (**A**) GR and (**C**) SOD, and enzyme activity with (**B**) GR and (**D**) SOD. The relative expression of genes was calculated by the comparative Ct method (2^−ΔΔCT^) using the 18s ribosomal protein as the internal reference gene. Each value represents the mean ± S.E.M. Letters indicate statistical differences among different treatments at the same time, statistical differences over time points. Symbols (*, #, %, and +) indicate statistical differences among the same treatments at different time points (two-way ANOVA, *p* < 0.05).

The GR gene expression was downregulated in all experimental groups and all kinetic experiments. The enzyme activity was similar to that of all groups compared to the control group. However, we should examine the same concentration over time. When we do this, we see an increase in its activity, even showing statistical differences (Figure 2A,B). The SOD gene expression presented the highest values at 3 and 6 h in all concentrations compared to the control group, showing statistical differences. Meanwhile, all groups’ enzyme activity presented similar values (Figure 2C,D).

### 3.2. Florfenicol (FLO)

**Figure 3:**
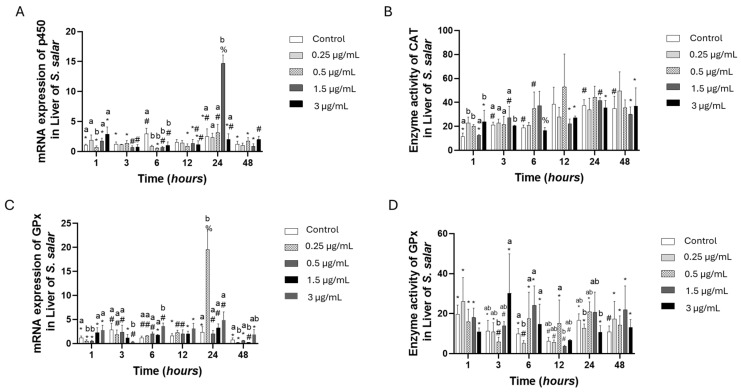
Liver cell culture treated with florfenicol “FLO”: gene expression with (**A**) Citochrome p450 and (**C**) GPx, and enzyme activity with (**B**) CAT and (**D**) GPx. The relative expression of genes was calculated by the comparative Ct method (2^−ΔΔCT^) using the 18s ribosomal protein as the internal reference gene. Each value represents the mean ± S.E.M. Letters indicate statistical differences among different treatments at the same time, statistical differences over time points. Symbols (*, #, and %) indicate statistical differences among the same treatments at different time points (two-way ANOVA, *p* < 0.05).

The p450 gene expression at 6 h presented downregulation in all experimental groups compared to the control group, while at hour 24, the 1.5 µg/mL of FLO was the highest value, almost five-fold compared to the control group. Meanwhile, catalase activity was similar to the control group (Figure 3A,B). The GPx gene expression presented the highest value at 24 h for 0.25 µg/mL of FLO, while at 48 h, the 0.25, 0.5, and 1.5 µg/mL concentrations of FLO presented the lowest values. Meanwhile, the enzyme activity presented some low activities at 6, 12, and 24 h for the control group (Figure 3C,D).

**Figure 4:**
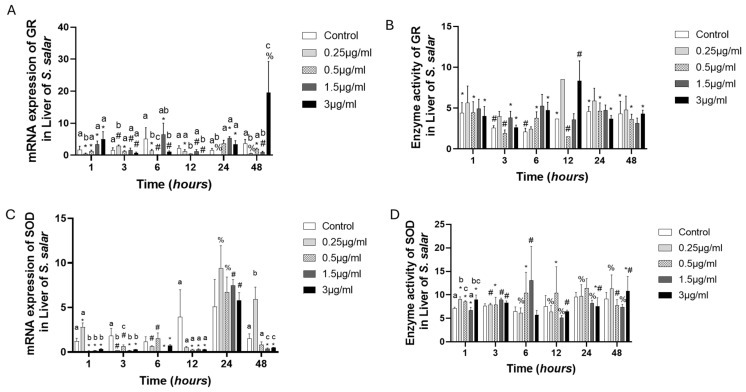
Liver cell culture treated with florfenicol “FLO”: gene expression with (**A**) GR and (**C**), SOD, and enzyme activity with (**B**) GR and (**D**) SOD. The relative expression of genes was calculated by the comparative Ct method (2^−ΔΔCT^) using the 18s ribosomal protein as the internal reference gene. Each value represents the mean ± S.E.M. Letters indicate statistical differences among different treatments at the same time, statistical differences over time points. Symbols (*, #, and %) indicate statistical differences among the same treatments at different time points (two-way ANOVA, *p* < 0.05).

The GR gene expression pattern was more or less clear, presenting downregulation in almost all experimental groups at all times, but 3 µg/mL of FLO at 48 h presented overexpression, eight-fold compared to the control group, while the GR enzyme activity values were similar to the control group. However, all experimental groups presented statistical differences for time (Figure 4A,B). The SOD gene expression presented two patterns: the first was at 1, 3, 6, and 12 h of exposure, where the expression was downregulated, while the second was at 24 h of exposure, where the experimental groups were similar to the control group. The enzyme activity presented statistical differences concerning time at the same concentration; at 1 h, there were statistical differences between different concentrations and the control group (Figure 4C,D).

Table 2 shows a summary of the results, indicating the main tendency of mRNA expression and enzymatic activity of liver cell culture challenged at OTC and FLO.

## 4. Discussion

Chile uses antibiotics heavily in fish farming, reaching 463 tons, with an annual consumption of 0.47 Kg of antibiotics per ton of farmed salmon; this use is making bacteria more resistant [26,36]. The OTC commercial dose given to salmonids in the diet is 75–100 mg/Kg [37]. Therefore, the commercial dose at plasma level is approximately 1 µg/mL. In the case of FLO, according to Martinsen et al. [25], the 10 mg/Kg commercial dose administered orally is equivalent to 4.41 µg/mL at plasma level at 12 h post-treatment. It is important to note that, according to Avendaño-Herrera et al. [36], Piscirickettsiosis is currently controlled by FLO incorporated into medicated feed. This is commonly administered as 20–30 mg/Kg for 14–21 days. Both precedents show that the plasma level dose of FLO for treatment of Piscirickettsiosis is between 8.82 and 13.23 µg/mL in plasma. Following these authors (Tafalla et al. [24]; Martinsen et al. [25]; Elema et al. [26]), we decided to determine the effect of both antibiotics on primary cultures of liver cells. It is known that the liver is the main metabolic organ and implies detoxification of the organism, as well as metabolic response [33,38]. Aquaculture is expanding and becoming more intensive, causing the use of disease control mechanisms such as antibiotics to be more common. This has an impact on the physiology of animals treated as in this study, where liver cells indicate responses to oxidative stress derived from treatment with the two most commonly used antibiotics in the industry.

Detoxification starts with cytochrome P450 [17,18], showing two patterns dependent on antibiotics in our results, with OTC presenting the highest expression at 6 h. However, initially and at the end of the experiment, downregulation was observed. At the same time, FLO at 6 h showed downregulation, indicating that patterns depend on antibiotics. These results are similar to those of a study on the green swordtail fish (*Xiphophorus helleri*) treated with the antibiotic norfloxacin. The antibiotic inhibited gene expression, although this inhibition was reversible and depended on the species’ sex; these results were more noticeable in females than males [39]. From the P450 point of view, this response could indicate eventual damage to the liver tissue; in our case, it would be damage to liver cells (as it was an in vitro experiment). As concluded, the study was carried out on broiler chickens subjected to different doses of florfenicol for 5 days [40]. The authors explain that the metabolites formed during the phase I biotransformation of florfenicol harm the liver. This causes liver injury, leading to apoptosis of these cells and loss of liver function, resulting in irreversible damage at the cellular level and, therefore, at the individual level [40].

Meanwhile, the catalase in both antibiotics presented a similar pattern without major changes; instead, a *Gadus morhua* “cod” study indicated that fish under the oral administration of FLO presented a downregulation of gene expression [41].

The low gene expression in GPx in OTC treatment may be due to a decrease in hydrogen peroxide (H_2_O_2_) levels to cope with the increase in ROS produced and thus repair the damage generated to prevent lipid peroxidation. This was observed at the enzymatic level in rainbow trout in an in vivo experiment, where the trout were exposed acutely (0.005, 0.050, 0.500, 5.000, and 50.00 mg/L) for 96 h and chronically (0.3125, 0.625, 1.25, 2.5, and 5.0 μg/L) for 28 days to oxytetracycline in the gills and liver. The authors observed an increase in GPx within 96 h of acute oxytetracycline exposure, while chronic exposure inhibited GPx activity [42]. Based on this, the GPx inhibition observed in our results is likely due to the high amount of free radicals in liver cells, indicating oxidative damage. Our study did not analyze this, but it gives us clues about how oxytetracycline might affect the antioxidant system [41]. Several studies in fish and mammals conclude that florfenicol inhibits GPx activity [41,43,44]. Interestingly, although various fluctuations were found in our results at the molecular level, low expression of the GPx gene was observed, which tells us that florfenicol inhibits GPx expression and enzymatic activity to a greater extent in *Salmo salar* liver cells, probably because it is not capable of breaking down hydrogen peroxide and lipid hydroperoxide.

The fluctuations observed in the results for oxytetracycline, with inhibition of glutathione reductase (GR) gene expression and virtually no changes in enzyme activity, indicate that oxytetracycline inhibits reactions that counteract ROS formation. The mechanism that would lead to the inhibition of these reactions is still unclear [45,46]; however, most studies establish that oxytetracycline is a promoter of ROS formation [9,12,13].

The enzyme GR is involved in the antioxidant defense mechanism by acting as a scavenger of oxygen radicals. It mitigates the oxidation of reduced glutathione (GSH) by promoting the transformation of oxidized glutathione (GSSG) back into its reduced form, GSH. This conversion ensures adequate levels of GSH, which are crucial for glutathione peroxidase (GPx) [45]. For florfenicol, the results are similar to those observed in a recent study conducted on rainbow trout, where the effects of florfenicol were evaluated at doses of 7.5 mg/Kg and 15 mg/Kg administered in the diet for 10 days, followed by 5 days without the antibiotic. This study observed no GR activity in trout exposed to florfenicol. However, it was observed after post-antibiotic treatment, indicating a delayed effect of the antibiotic. Along with this, an increase in glutathione was recorded as the highest concentration of florfenicol, which remained unchanged even after post-antibiotic treatment, as did the total glutathione concentration. As mentioned earlier, GR activity catalyzes the regeneration of GSH from GSSG. Therefore, the increase in glutathione makes sense just before observing a response in glutathione reductase in post-antibiotic fish, indicating that the antioxidant system is coping with the toxicity generated by the florfenicol doses [7].

SODs are a group of metalloenzymes that perform a crucial function as antioxidants and are part of the defense against the toxic effects generated by superoxide radicals (O_2_^−^) in aerobic organisms [47]. A study conducted on rainbow trout (*Oncorhynchus mykiss*) showed that the administration of oxytetracycline significantly decreased SOD activity in the liver [42]. Similar results were observed in rainbow trout (*O. mykiss*). However, when looking at the effects of propolis on oxytetracycline, they obtained a significant decrease in SOD activity [41]. These data obtained from rainbow trout do not agree with the results of our in vitro study, where an increase in SOD expression was observed at the mRNA level. However, enzymatic activity increased only at the highest doses at 12 and 48 h. This would indicate an activation of the antioxidant system due to the formation of oxygen radicals produced by oxytetracycline. SOD would prevent lipid peroxidation, thereby preventing the formation of free radicals and thus avoiding chronic oxidative stress in *Salmo salar*, which could cause permanent damage to liver cells [48]. Lipid peroxidation is considered the initial step in cell membrane damage caused by pesticides, metals, or xenobiotics since free radicals capture electrons from the lipids that make up the cell membrane, causing oxidative lipid degradation [49]. Free radicals attack the cell membrane, destabilizing it and even disintegrating it. Consequently, this cascading effect would cause muscle degradation, hemolysis, and nervous system and metabolic deterioration, causing multisystem damage that can lead to cell death [50,51].

During treatment with florfenicol, SOD showed inhibition of this antioxidant in the first 12 h and at 48 h at the highest doses, probably due to an increase in lipid peroxidation, since inhibition of endogenous antioxidants such as SOD accompanies it. This was observed in a nephrotoxicity study in chickens, where the authors mentioned the frequent accumulation of this antibiotic in different tissues, which caused lipid peroxidation, resulting in an inhibition of SOD [39]. Therefore, florfenicol would cause serious damage to liver cells, mainly in the plasma membrane, by damaging its structure through lipid peroxidation, leading to irreversible cellular damage in *Salmo salar*.

For mammals, there have been important advances in our conceptual understanding of how antibiotics function and how to improve their use. Different classes of bactericidal antibiotics, regardless of their drug target interactions, generate varying levels of deleterious reactive oxygen species (ROS) that contribute to cell death [52,53]. Rijkers et al. [54], Siwicki et al. [55], and Guardiola et al. [56] have shown that oxytetracycline suppresses immune functions in carp, rainbow trout, and Seabream.

Additionally, florfenicol does not significantly influence the studied immune parameters, although a slight effect on phagocyte function has been observed [56]. Oxytetracycline is an ecotoxic chemical that increases the environmental risk of affecting non-target organisms [57]. Recently, Nazari et al. [58] demonstrated the effects of oxytetracycline on liver enzyme activity and oxidative stress indexes in rainbow trout. Tetracycline antibiotics are known to enhance ROS production [12]. With this study, we are advancing our understanding of the effects of antibiotics on fish, especially those important for aquaculture, such as *Salmo salar*.

## 5. Conclusions

In recent years, several studies have explored the toxicity of antibiotics such as oxytetracycline (OTC) and florfenicol (FLO) in fish, primarily assessing general physiological parameters or liver tissue damage. However, most of these studies have focused on whole organisms rather than in vitro cellular models. Research on liver cell cultures or primary fish cultures has been scarce, and even more limited is information on the molecular and enzymatic responses associated with the oxidative stress induced by these antibiotics. In terms of innovation, this study is pioneering in evaluating, using time-based kinetics, the response to oxidative stress in primary cultures of *Salmo salar* liver cells to different concentrations of OTC and FLO, integrating two approaches: both gene expression and the enzymatic activity of key markers. This provides a more accurate and early view of the cellular damage that these antibiotics can induce in aquaculture.

In summary, our results indicate that both antibiotics affect the oxidative response in liver cells, ranging from transcriptional expression to enzymatic activity. They generated “upregulation” and “downregulation” responses at the beginning and end of the experiment, with dose dependence and time being the factors that influenced the change. These studies are helping to clarify the effect of antibiotics on physiological responses, to achieve better aquaculture management.

## Figures and Tables

**Table 2:** Summary of all the responses evaluated with both antibiotics, mRNA expression and enzymatic activity. Black arrows show as the down or up regulation/activities. Red or blue arrows indicate how was the average of expression or activity.

	OTC	FLO
Parameter	Liver	Liver
expression		
**cat**	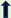	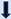
**sod**	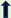	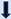
**gpx**	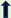	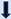
**gr**	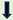	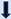
*overall*	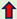	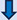
activity		
**CAT**	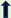	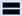
**SOD**	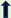	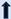
**GPX**	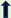	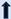
**GR**	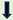	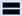
*overall*	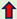	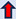

## Data Availability

Data Availability Statements are available by requirement.
